# Clinical Characteristics and Survival Trends of Male Breast Cancer in the United States: A Propensity Score Matched Analysis †

**DOI:** 10.3390/jpm15070321

**Published:** 2025-07-17

**Authors:** Jayasree Krishnan, Malak Alharbi, Kristopher Attwood, Arya Mariam Roy

**Affiliations:** 1 Division of Oncology, Department of Medicine, Roswell Park Comprehensive Cancer Center, Buffalo, NY 14203, USA; 2 Department of Internal Medicine, King Abdul-Aziz University, Jeddah, Saudi Arabia; 3 Department of Biostatistics, Roswell Park Comprehensive Cancer Center, Buffalo, NY 14203, USA; 4 Division of Medical Oncology, Department of Medicine, The Ohio State University Comprehensive Cancer Center, College of Medicine, The Ohio State University Wexner Medical Center, Columbus, OH 43210, USA

**Keywords:** male breast cancer, survival trend, overall survival, female breast cancer

## Abstract

**Background:** Male breast cancer (MBC) is extremely rare, representing less than 1% of breast cancer (BC). Owing to the rarity, there is a substantial knowledge gap regarding the survival trends of MBC compared with female breast cancer (FBC). **Methods:** We queried the National Cancer Database for BC patients diagnosed during 2004–2018 and utilized an inverse propensity weighted cox regression model assessed the association between sex and overall survival (OS) and survival trends over time by sex. **Results:** We identified 24,055 MBC and 2,532,470 FBC patients. Patients with MBC were older (mean age: 65.6 vs. 61.4 years), and more likely to have stage IV at diagnosis (7% vs. 4.7%), larger tumors (cT4: 6% vs. 3.7%), and node-positive disease (18.5% vs. 15.5%) (*p* < 0.001) compared with FBC. MBC were more likely to be estrogen (ER) (88.5% vs. 78.5%) and progesterone receptor (PR) (79.6% vs. 68%) positive and less likely to be HER2 receptor positive (7.9% vs. 9.3%) or triple negative (2.8% vs. 7.6%) compared with FBC (all *p* < 0.001). The OS rates were lower in MBC compared with FBC (5-year: 73% vs. 83%; 10-year: 54% vs. 70%, *p* < 0.001). In the propensity weighted cox-regression model, males had higher mortality than females with BC (HR 2.8, 95% CI 2.88–2.9, *p* < 0.001). The 5-year OS rates increased steadily for FBC from 2004–2015; however, the survival rates did not improve for MBC over the last decade. **Conclusions**: Our study shows that MBC patients continue to have poor OS compared with patients with FBC and no significant improvement in survival of MBC patients over the past decade. These results underscore the need to investigate personalized treatment interventions for patients with MBC to improve outcomes.

## 1. Introduction

Male breast (MBC) cancer is a rare entity, accounting for less than 1% of cancers in men globally [1]. In 2025, an estimated 2800 men and 316,950 women would be diagnosed with breast cancer (BC) in the United States (US) [2]. Older age, obesity, excessive alcohol consumption, radiation exposure, hyperestrogenism due to Kleinfelter syndrome, and BRCA2 mutation have been identified as risk factors for MBC [3,4,5]. Studies have shown that patients with MBC are usually diagnosed at older age and at a more advanced stage and more likely to have estrogen receptor and/or progesterone receptor positive disease compared with female breast cancer (FBC) patients [5,6,7,8,9,10,11].

Due to the rarity of MBC, there are limited prospective clinical trials investigating treatments in this specific population [4]. Consequently, treatment for patients with MBC has historically been extrapolated and adapted based on data from patients with FBC [4,12,13,14] despite evidence suggesting that MBC has a distinct biological characteristic compared with FBC [15,16,17,18]. Over the past decade, the incidence of breast cancer has increased, and early diagnosis through mammograms, along with improved treatment strategies, has led to improved breast cancer survival rates among women [19]. Few studies have examined the survival rates among patients with MBC compared with FBC, with conflicting results. While some studies have reported worse survival rates [8,20,21,22], others have found better or similar survival rates among patients with MBC [23,24,25]. Gwark et al., have reported that younger (≤40 years) MBC patients had a better 5-year overall survival than the older patients [26]. A recent study by Leone et al., reported no statistically significant improvement in BC-specific survival rates among patients with MBC over the past 3 decades [27]. However, the survival trends for patients with MBC over the past decade compared with FBC remain unknown.

In this study, we used data from the National Cancer Database (NCDB) to investigate the differences in clinical and demographic characteristics between patients with MBC and FBC diagnosed between 2004 and 2018. We also compared the overall survival (OS) of patients with MBC and FBC by stage and tumor subtypes and analyzed the survival trends in the last decade.

## 2. Materials and Methods

### 2.1. Data Source, Patient Selection, and Variables

**Figure 1:**
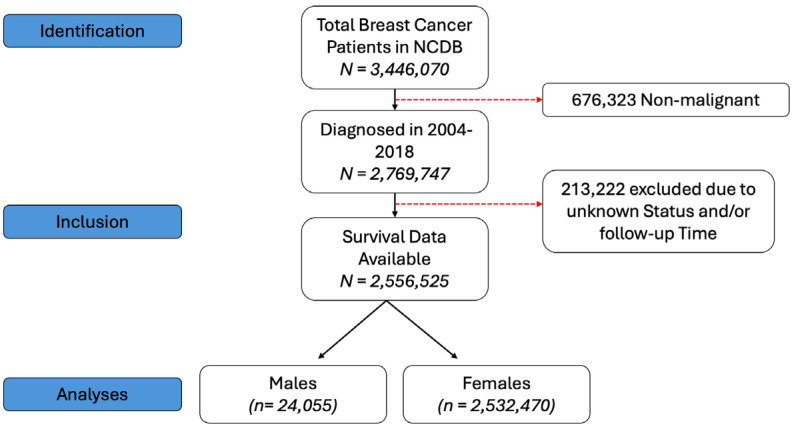
Study design for patient selection.

The National Cancer Database (NCDB) is a joint program from the Commission on Cancer of the American College of Surgeons and American Cancer Society and is a nationwide oncology outcomes database that collects data on approximately 70% of all new invasive cancer diagnoses in the US. In this cohort study, we included patients aged 18 years or older with BC (American Joint Cancer Committee clinical stage I–IV disease) that was diagnosed from 2004 to 2018. Patients were excluded if there were any missing data on follow-up information, survival or gender. As the data are publicly available and de-identified to ensure anonymity, our study was exempted from institutional review board approval, and informed consent was also waived for the same reason. Our study follows the Strengthening the Reporting of Observational Studies in Epidemiology (STROBE) reporting guidelines. The study design is shown in Figure 1.

The demographic (age, sex, race, and insurance), clinicopathologic (clinical TNM stage, grade, estrogen receptor (ER), progesterone receptor (PR), HER2 status, year of diagnosis), treatment (time to treatment, chemotherapy, hormonal therapy, and radiotherapy) characteristics, and survival variables of patients were collected. The primary outcome of the study was OS, which was calculated from the date of the diagnosis until death due to any cause or last contact. Patients without a recorded death event were censored for OS at their last contact date.

### 2.2. Statistical Analysis

The demographic, clinical, and treatment characteristics were summarized by sex (males vs. female) in the overall sample, and within stage (I–III, IV) groups using mean, median, standard deviation, and range for continuous variables and frequencies and relative frequencies for categorical variables. Statistical comparisons were made using Mann–U Whitney test, or Fisher exact test wherever appropriate.

OS of patients with MBC and FBC were summarized using standard Kaplan–Meier methods. Five- and ten-year survival rates with 95% confidence intervals were calculated for the overall sample and by the subgroups mentioned above and compared using log rank tests.

Univariate cox regression modeling was used to assess associations between sex and OS. A propensity weighted cox regression modeling was used to assess the association between sex and overall survival, in order to control potential confounders and selection bias. Logistic regression models with sex as the outcome, and age, race, insurance, T-stage, N-stage, M-stage, subtype, grade, chemotherapy, radiation, hormone therapy, and time to definitive treatment as predictor variables were used to find the predicted probabilities. Models were fit using firth’s method. Propensity weights by sex (males: w = 1/prob, females: w = 1/(1 − prob)) were generated and observations where a male subject had a predicted probability greater than the maximum probability from the females was excluded. Similarly, observations where a female subject had a predicted probability less than the minimum probability from the males were also excluded. Hazard ratios (HRs) for the sex effect (MBC vs. FBC) and their associated 95% confidence intervals (CIs) were obtained from the Cox regression analysis. Five-year survival rates for males and females were measured by year of diagnosis from 2004–2015 to study the survival trend. A *p* value below 0.05 was regarded as statistically significant.

## 3. Results

### 3.1. Baseline Characteristics

A total of 24,055 patients with MBC and 2,532,470 with FBC were identified. The demographic, clinical and treatment characteristics of the overall sample by sex are summarized in Table 1. Patients with MBC were older at diagnosis (median age: 66 vs. 62 years), had higher frequency of Blacks (12.8 vs. 11.2%) and more likely to have government insurance (55.7% vs. 45.8%) compared with those with FBC (all *p* < 0.001). In addition, more patients with MBC had stage IV (7% vs. 4.7%), larger tumors (cT4: 6% vs. 3.7%), and node positive disease at diagnosis (18.5% vs. 15.5%) compared with patients with FBC (all *p* < 0.001). Moreover, MBC patients were more likely ER (88.5% vs. 78.5%) and PR (79.6% vs. 68%) positive and less likely HER2 receptor positive (7.9% vs. 9.3%) or triple negative (2.8% vs. 7.6%) compared with FBC (all *p* < 0.001). Interestingly, patients with MBC were less likely to receive treatment with chemotherapy (38.7% vs. 41.2%), radiation therapy (32% vs. 52.6%), and hormonal therapy (59.8% vs. 62.5%) compared with patients with FBC (*p* < 0.001). We observed a steady and similar rate of increase in the incidence of breast cancer from 2004 to 2018 in both the groups. The demographic, clinical and treatment characteristics of the subgroups stage I–III and stage IV by sex are summarized in Supplementary Tables S1 and S2 respectively.

### 3.2. Overall Survival

#### 3.2.1. Overall Survival by Tumor Stage

**Figure 2:**
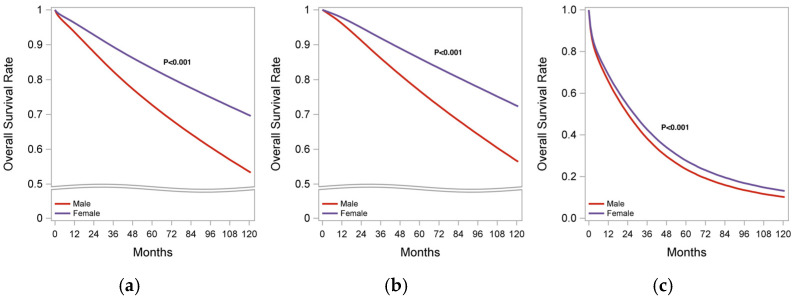
Kaplan–Meier survival analysis of MBC and FBC patients. (**a**) Overall cohort; (**b**) early stage (stages I–III); (**c**) stage IV BC.

The median follow-up in the overall cohort was 84.5 months (84.5 months in FBC vs. 82.8 months in MBC). The 5-year and 10-year OS rates by sex in the overall cohort are summarized in Table 2. The survival rates in stages 1–3 and stage 4 subgroups are presented in Supplementary Tables S3 and S4, respectively. The Kaplan–Meier analysis by sex for the overall cohort, as well as for stages I–III and stage IV, is illustrated in Figure 2. We defined early stage as stages I–III at diagnosis.

In the overall cohort, patients with MBC exhibited a significantly lower OS rate compared with FBC (5-year OS: 73% vs. 83%; 10 yr OS: 54% vs. 70%; *p* < 0.001). Propensity weighted analysis showed that patients with MBC had a higher risk of mortality (HR 2.89, 95% CI:1.88–2.90; *p* < 0.001) compared with FBC. These findings remained consistent when analyzed by stage, both in stages I–III and stage IV, as detailed in Supplementary Tables S3 and S4, respectively.

#### 3.2.2. Overall Survival by Tumor Subtype

The 5-year and 10-year OS rates by tumor subtypes in the overall cohort are summarized in Table 2. The three tumor subtypes included are TNBC, HER2+BC and HR+/HER2- BC, where HR+ is defined as ER or PR positive. Patients with MBC exhibited a lower OS rate compared with FBC across all tumor subtypes, TNBC (5-yr OS: 61% vs. 74%; 10-yr OS: 57% vs. 64%; *p* < 0.001), HER2+BC (5-yr OS: 71% vs. 84%; 10-yr OS: 54% vs. 72%; *p* < 0.001), and HR+/HER2- BC (5-yr OS: 75% vs. 86%; 10-yr OS: 55% vs. 72%; *p* < 0.001). Propensity weighted regression analysis showed that MBC patients with TNBC (HR 1.22, CI 1.21–1.24; *p* < 0.0001) and HER2+ BC (HR 2, CI 1.98–2.03; *p* < 0.0001) had a higher risk of mortality compared with FBC; however, MBC patients with HR+/HER2-BC (HR 0.78, CI 0.78–0.79; *p* < 0.0001) had a lower risk of mortality compared with FBC after adjusting for confounding variables.

#### 3.2.3. Survival Trends

**Figure 3:**
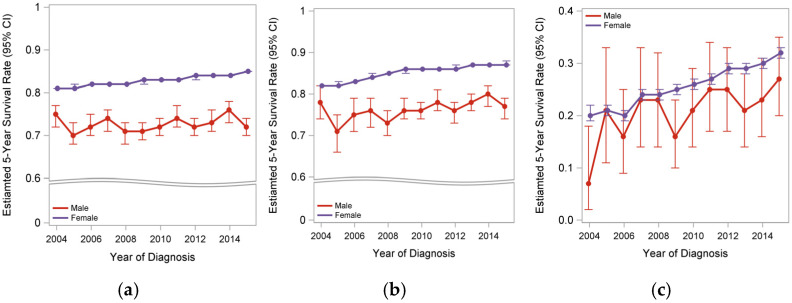
Survival trends of MBC and FBC patients between 2004–2014. (**a**) Overall cohort; (**b**) early stage (stages I–III); (**c**) stage IV BC.

The survival trends for patients with MBC and FBC from 2004 to 2015 are shown in Figure 3. Overall, the survival rate among FBC patients has improved; however, no significant improvement was observed in the survival rate of patients with MBC over the past decade. Similar disparate trends were noted in early-stage MBC (stages I–III), although some improvement in survival was seen among patients with stage IV MBC over time (Figure 3).

## 4. Discussion

Our study shows that the incidence of male and female BC has increased from 2004–2018 at a similar rate. However, patients with MBC continued to have poor OS compared with patients with FBC. Interestingly, male patients with early-stage HR-positive BC exhibited lower risk of death compared with FBC in multivariable analyses after adjusting for confounders. Over the past decade, the survival trends of patients with FBC have improved over time, however no significant improvement has been noted in the overall survival of MBC patients.

Our study results showing MBC patients with worse survival outcomes compared with FBC are consistent with prior published literature [8,20,21,22,28]. Grief et al. utilized data from NCDB from 1998–2007 and reported that men with BC had significantly poor OS when compared with women, specifically for patients with early-stage BC [20]. Another NCDB-based study (2004–2014) by Wang et al. found that male patients with BC had a 19% higher overall mortality compared with their female counterparts, with clinical characteristics and undertreatments contributing to 63% of the excess mortality noted among male BC patients [21]. Our updated study, evaluating patients diagnosed until 2018, shows similar disparate trends in survival, and these findings taken together suggest that the progress in improving the survival of MBC patients is significantly lacking over decades, which highlights the need to focus on research on the tumor biology of MBC patients and evaluation for personalized treatment options.

Few studies have analyzed the survival trends in MBC and FBC over time. Anderson et al. have reported that the survival rates have improved over time for both MBC and FBC but that the progress in men is lagging [8]. A recent study by Leone et al., reported improved OS in men consistent with increasing life expectancy; however, no statistically significant improvement in breast-cancer-specific survival rates were noted among MBC patients over the past three decades [27]. Our study is the first to report the survival trends in the past decade compared with FBC. We found that the OS has not improved significantly in men despite advances in breast cancer treatment options and improved survival in female counterparts. Interestingly, while MBC patients had a lower OS in the unadjusted population level trends, patients with early-stage HR-positive MBC had a lower risk of death compared with FBC in multivariable analysis. The adjusted analysis highlights that, when controlling for baseline characteristics and treatment factors, early-stage HR-positive MBC patients may fare slightly better than FBC patients, possibly due to differences in tumor biology, treatment response, adherence, comorbidity profiles or other unmeasured confounders. Prior studies have demonstrated high rates of recurrence in HR-positive MBC compared with FBC [25], which could explain the poor OS seen in MBC in our study, along with prior studies [22,29,30]. Future studies are needed to understand the lower risk of death in MBC patients with early-stage HR-positive BC.

The overall poor survival in MBC patients could partly be explained by later stage and older age at diagnosis in men; however, the differences persisted even after adjustment for age and staging. Poor compliance to hormonal therapy due to adverse effects could impact treatment outcomes in men [31,32]; however, recent studies have shown similar rates of discontinuation and adherence rate between both groups [33,34]. A study by Venigalla et al., reported the underutilization of adjuvant hormonal therapy in males with HR-positive BC, despite the survival benefits associated with its use [35]. Such disparate trends in the utilization of therapies and compliance issues could explain the observed lag in survival between males and females with BC, in addition to potential biological and gender-specific differences in tumor responses. There is a lack of randomized controlled trials evaluating the efficacy of hormone therapy and chemotherapy in MBC patients and future translational research focusing on understanding molecular differences, tumor biology in MBC, and treatment efficacy is warranted to improve outcomes. The limited improvement in survival and mortality rates among patients with MBC over the years, despite advances in treatment options, underscores the need for personalized therapies based on a deeper understanding of tumor biology and treatment responses.

One of the strengths of our study is the inclusion of a large and diverse patient cohort, allowing for a thorough analysis of gender disparity while adjusting for several covariates. Our study also has several limitations. First, there are inherent selection biases owing to the retrospective nature of the study design. Data on specific treatment regimens including the dose, and duration of chemotherapy, are not available in NCDB. In addition, there is also a lack of data on the cause of death, specifically breast-cancer-specific mortality, presence of genetic mutations (e.g., BRCA), treatment side effects, disease status such as relapse and/or recurrence of disease. Lastly, data on comorbidity, patient compliance, and socioeconomic factors which can affect the access to care and clinical outcomes of these patients are missing.

## 5. Conclusions

MBC patients have higher mortality rates and poor clinical outcomes compared with FBC patients. We demonstrate that the survival of MBC patients has not improved over the last decade, even after adjusting for clinicopathological and treatment characteristics. These findings suggest the need to investigate personalized treatment interventions for male breast cancer patients, especially given the disparate trends in survival among male and female BC over a period of time.

## Figures and Tables

**Table 1:** Demographic and clinical characteristics of overall population by gender.

Characteristics	Male (n = 24,055)	Female (n = 2,532,470)	Overall (N = 2,556,525)	*p*-Value
**Age at diagnosis**				
Median (IQR), years	66.0 (18.0–90.0)	62.0 (18.0–90.0)	62.0 (18.0–90.0)	<0.01
**Race**				
White	19,952 (82.9%)	2,107,070 (83.2%)	2,127,022 (83.2%)	<0.01
Black	3018 (12.8%)	284,748 (11.2%)	287,829 (11.2%)	
Asian	355 (1.5%)	67,600 (2.7%)	67,955 (2.7%)	
Other	407 (1.7%)	47,936 (1.9%)	48,343 (1.9%)	
N/R	260 (1.1%)	25,116 (1.0%)	25,376 (1.0%)	
**Insurance status**				
Uninsured	550 (2.3%)	50,340 (2.0%)	50,890 (2.0%)	<0.01
Private	9612 (40.0%)	1,275,361 (50.3%)	1,284,973 (50.3%)	
Governmental	13,401 (55.7%)	1,158,620 (45.8%)	1,172,021 (45.8%)	
N/R	492 (2.0%)	48,149 (1.9%)	48,641 (1.9%)	
**ER status**				
Negative	1636 (6.8%)	461,310 (18.2%)	462,946 (18.1%)	<0.01
Positive	21,283 (88.5%)	1,987,839 (78.5%)	2,009,122 (78.6%)	
N/R	1136 (4.7%)	83,321 (3.3%)	84,457 (3.3%)	
**PR status**				
Negative	3599 (15.0%)	713,689 (28.2%)	717,288 (28.1%)	<0.01
Positive	19,138 (79.5%)	1,722,787 (68.0%)	1,741,925 (68.1%)	
N/R	1318 (5.5%)	95,994 (3.8%)	97,312 (3.8%)	
**HER2 status**				
Negative	12,762 (53.1%)	1,352,184 (53.4%)	1,364,946 (53.4%)	<0.01
Positive	1902 (7.9%)	236,113 (9.3%)	238,015 (9.3%)	
N/R	9391 (39.0%)	944,173 (37.3%)	953,564 (37.3%)	
**Subtype**				
TNBC	664 (2.8%)	191,589 (7.6%)	192,253 (7.5%)	<0.01
HER2+	1902 (7.9%)	236,113 (9.3%)	238,015 (9.3%)	
ER/PR+ and HER2-	12,072 (50.2%)	1,158,886 (45.8%)	1,170,958 (45.86%)	
N/R	9417 (39.1%)	945,882 (37.4%)	955,299 (37.4%)	
**Grade**				
Well differentiated	3161 (13.2%)	531,408 (21.1%)	534,569 (21.1%)	<0.01
Moderately differentiated	11,143 (46.7%)	1,024,204 (40.9%)	1,035,347 (40.9%)	
Poorly differentiated	7300 (30.6%)	741,683 (29.5%)	748,983 (29.5%)	
Undifferentiated	88 (0.4%)	8389 (0.3%)	8477 (0.3%)	
N/R	2181 (9.1%)	206,997 (8.2%)	209,178 (8.2%)	
**Stage at Diagnosis**				
0	254 (1.1%)	54,577 (2.1%)	54,831 (2.1%)	<0.01
1	8033 (33.4%)	1,133,531 (44.8%)	1,141,564 (44.8%)	
2	6779 (28.2%)	561,502 (22.2%)	568,281 (22.2%)	
3	1670 (6.9%)	149,746 (5.9%)	151,416 (5.9%)	
4	1680 (7.0%)	119,217 (4.7%)	120,897 (4.7%)	
N/R	5639 (23.4%)	513,897 (20.3%)	519,536 (20.3%)	
**Tumor stage at diagnosis**				
cT1	8881 (37.0%)	1,215,583 (47.9%)	1,224,464 (47.9%)	<0.01
cT2	6962 (28.9%)	535,535 (21.2%)	542,497 (21.2%)	
cT3	749 (3.1%)	111,741 (4.4%)	112,490 (4.4%)	
cT4	1454 (6.0%)	94,742 (3.8%)	96,196 (3.8%)	
N/R	6009 (25.0%)	574,869 (22.7%)	580,878 (22.7%)	
**Nodal staging a diagnosis**				
cN0	14,240 (59.2%)	1,655,634 (65.3%)	1,669,874 (65.3%)	<0.01
cN1+	4453 (18.5%)	391,812 (15.5%)	396,265 (15.5%)	
N/R	5362 (22.3%)	485,024 (19.2%)	490,386 (19.2%)	
**Metastatic stage at diagnosis**				
cM0	21,317 (88.6%)	2,329,909 (92.0%)	2,351,226 (92.0%)	<0.01
cM1	1677 (7.0%)	118,192 (4.7%)	119,869 (4.7%)	
N/R	1061 (4.4%)	84,369 (3.3%)	85,430 (3.3%)	
**Average tumor size**				
(IQR) in mm	20.0 (0.0–989.0)	16.0 (0.0–989.0)	16.0 (0.0–989.0)	<0.01
**Average time to definitive treatment/surgery** **(IQR) in months**	21.0 (0.0–111.0)	27.0 (0.0–5668.0)	27.0 (0.0–5668.0)	<0.01
**Chemotherapy**				
Yes	9316 (38.7%)	1,043,280 (41.2%)	1,052,596 (41.2%)	<0.01
No	12,025 (50.0%)	1,249,471 (49.3%)	1,261,496 (49.3%)	
N/R	2714 (11.3%)	239,719 (9.5%)	242,433 (9.5%)	
**Hormonal therapy**				
Yes	14,388 (59.9%)	1,582,641 (62.5%)	1,597,029 (62.5%)	<0.01
No	7226 (30.0%)	724,600 (28.6%)	731,826 (28.6%)	
N/R	2441 (10.1%)	225,229 (8.9%)	227,670 (8.9%)	
**Radiation Therapy**				
Neoadjuvant	82 (0.3%)	8746 (0.3%)	8828 (0.3%)	<0.01
Adjuvant RT	7686 (32.0%)	1,333,198 (52.6%)	1,340,884 (52.6%)	
No RT	15,307 (64.0%)	1,102,466 (43.6%)	1,117,773 (43.6%)	
Both neo/adjuvant RT	12 (0.0%)	1274 (0.1%)	1286 (0.1%)	
N/R	968 (4.0%)	86,786 (3.4%)	87,754 (3.4%)	
**Year of diagnosis**				
2004	1181 (4.9%)	129,657 (5.1%)	130,838 (5.1%)	0.09
2005	1222 (5.1%)	133,756 (5.3%)	134,978 (5.3%)	
2006	1384 (5.8%)	139,566 (5.5%)	140,950 (5.5%)	
2007	1465 (6.1%)	146,180 (5.7%)	147,645 (5.7%)	
2008	1462 (6.1%)	152,932 (6.0%)	154,394 (6.0%)	
2009	1473 (6.1%)	159,612 (6.3%)	161,085 (6.3%)	
2010	1602 (6.7%)	159,098 (6.3%)	160,700 (6.3%)	
2011	1579 (6.6%)	167,863 (6.6%)	169,442 (6.6%)	
2012	1634 (6.8%)	172,802 (6.8%)	174,436 (6.8%)	
2013	1704 (7.1%)	180,239 (7.1%)	181,943 (7.1%)	
2014	1722 (7.2%)	186,494 (7.4%)	188,216 (7.4%)	
2015	1885 (7.8%)	192,533 (7.6%)	194,418 (7.6%)	
2016	1859 (7.7%)	197,364 (7.8%)	199,223 (7.8%)	
2017	1921 (8.0%)	204,375 (8.1%)	206,296 (8.1%)	
2018	1962 (8.2%)	209,999 (8.3%)	211,961 (8.4%)	

IQR: interquartile range; N/R: not reported; ER: estrogen receptor; PR: progesterone receptor; TNBC: triple negative breast cancer; HER2: human epidermal growth factor receptor 2; cT1: clinical staging T1; cN0: clinical nodal stage 0; cM0: no distant metastasis via clinical assessment; cM1: distant metastasis is present via clinical assessment; RT: radiation therapy.

**Table 2:** Survival analysis in overall cohort and by tumor subtypes.

Cohort	Gender	Survival Rate		*p*-Value	Univariate ^a^	Multivariate ^b^	*p*-Value
		5 yr. (95% CI)	10 yr. (95% CI)		HR^#^ (95% CI)	HR^#^ (95% CI)	
Overall cohort	Female	0.83 (0.83, 0.83)	0.70 (0.70, 0.70)	<0.001	Ref	Ref	<0.0001
	Male	0.73 (0.72, 0.73)	0.54 (0.53, 0.55)		1.73 (1.70–1.77)	2.89 (2.88–2.90)	
TNBC	Female	0.74 (0.74, 0.74)	0.64 (0.63, 0.64)	<0.001	Ref	Ref	<0.0001
	Male	0.61 (0.57, 0.65)	0.57 (0.52, 0.61)		1.64 (1.44–1.86)	1.22 (1.21–1.24)	
HER2+ BC	Female	0.84 (0.84, 0.84)	0.72 (0.72, 0.73)	<0.001	Ref	Ref	<0.0001
	Male	0.71 (0.69, 0.73)	0.54 (0.50, 0.59)		1.95 (1.79–2.12)	2.00 (1.98–2.03)	
HR+/HER2-BC	Female	0.86 (0.86, 0.86)	0.72 (0.71, 0.72)	<0.001	Ref	Ref	<0.0001
	Male	0.75 (0.74, 0.76)	0.55 (0.53, 0.57)		1.91 (1.85–1.98)	0.78 (0.78–0.79)	

Note: TNBC; triple negative breast cancer, HR; hormone receptor, CI; confidence interval, HR^#^; hazard ratio, ^a^ univariate logistic regression, ^b^ propensity weighted cox regression model.

## Data Availability

The dataset, the National Cancer Database, is publicly available through the American College of Surgeons https://www.facs.org/quality-programs/cancer/ncdb (accessed on 1 July 2023). Specific data used for this study are available from the authors upon reasonable request.

## Supplementary Materials

The following supporting information can be downloaded at https://www.mdpi.com/article/10.3390/jpm15070321/s1. Table S1: Demographic and Characteristics of Stage I, II and III population by Gender; Table S2: Demographic and Clinical Characteristics of stage IV population by gender; Table S3: Survival analysis in stage I-III subgroup and by tumor subtypes; Table S4: Survival analysis in stage IV subgroup and by tumor subtypes.
