# Supplementary material for: Clinical Characteristics and Survival Trends of Male Breast Cancer in the United States: A Propensity Score Matched Analysis
Source: J Pers Med. 2025 Jul 17;15(7):321. doi: 10.3390/jpm15070321 (PMC12299033; doi:10.3390/jpm15070321)
Supplement: Supplementary file 1 [file jpm-15-00321-s001.zip › jpm-3656826-supplementary.pdf]

Supplementary Table S1. Demographic and Clinical Characteristics of Stage I, II, and III population by Gender				
**Characteristics**	**Male (n=16,482)**	**Female (n=1,844,779)**	**Overall (N=1,861,261)**	**P-value**
**Age at diagnosis**<br>Median (IQR), years	66.0 (18.0 - 90.0)	62.0 (18.0 - 90.0)	62.0 (18.0 - 90.0)	<0.01
**Race**				
White	13,757 (83.5%)	1,538,357 (83.4%)	1,552,114 (83.4%)	
Black	2,064 (12.5%)	204,149 (11.1%)	206,213 (11.1%)	
Asian	239 (1.5%)	50,288 (2.7%)	50,527 (2.7%)	<0.01
Other	267 (1.6%)	35,721 (1.9%)	35,988 (1.9%)	
N/R	155 (0.9%)	16, 264 (0.9%)	16,419 (0.9%)	
**Insurance status**				
Uninsured	326 (2.0%)	33,357 (1.8%)	33,683 (1.8%)	
Private	6,577 (39.9%)	931,267 (50.5%)	937,844 (50.4%)	
Governmental	9,267 (56.2%)	847,876 (46.0%)	857,143 (46.1%)	<0.01
N/R	312 (1.9%)	32,279 (1.7%)	32,591 (1.7%)	
**ER Status**				
Negative	1,032 (6.3%)	329,016 (17.8%)	330,048 (17.7%)	<0.01
Positive	15,002 (91.0%)	1,481,737 (80.3%)	1,496,739 (80.4%)	
N/R	448 (2.7%)	34,026 (1.9%)	34,474 (1.9%)	
**PR status**				
Negative	2,295 (13.9%)	507,378 (27.5%)	509,673 (27.5%)	<0.01
Positive	13,660 (82.9%)	1,296,696 (70.3%)	1,310,356 (70.3%)	
N/R	527 (3.2%)	40,705 (2.2%)	41,232 (2.2%)	
**HER2 status**				
Negative	10,332 (62.7%)	1,156,796 (62.7%)	1,167,128 (62.7%)	<0.01
Positive	1,459 (8.9%)	190,156 (10.3%)	191,615 (10.3%)	
N/R	4,691 (28.4%)	497,827 (27.0%)	502,218 (27.0%)	
**Subtype**				
TNBC	511 (3.1%)	163,276 (8.9%)	163,787 (8.9%)	
HER2+	1,459 (8.9%)	190,156 (10.3%)	191,615 (10.3%)	<0.01
ER/PR+ and HER2-	9,807 (59.5%)	992,572 (53.8%)	1,002,379 (53.8%)	
N/R	4,705 (28.4%)	498,775 (27.0%)	503,480 (27.0%)	
**Grade**				
Well differentiated	2,305 (14.1%)	407,311 (22.2%)	409,616 (22.2%)	
Moderately differentiated	8,021 (49.0%)	772,892 (42.2%)	780,913 (42.2%)	<0.01
Poorly differentiated	5,036 (30.7%)	542,882 (29.6%)	547,918 (29.6%)	
Undifferentiated	31 (0.2%)	4,263 (0.2%)	4,294 (0.2%)	
N/R	990 (6.0%)	106,897 (5.8%)	107,887 (5.8%)	
**Overall stage**				
I	8,033 (48.8%)	1,133,531 (61.4%)	1,141,564 (61.4%)	
II	6,779 (41.1%)	561,502 (30.5%)	568,281 (30.5%)	<0.01
III	1,670 (10.1%)	149,746 (8.1%)	151,416 (8.1%)	

**Tumor stage at diagnosis**				
cT1	8,449 (51.3%)	1,173,906 (63.6%)	1,182,355 (63.6%)	
cT2	6,225 (37.8%)	492,599 (26.7%)	498,824 (26.7%)	<0.01
cT3	597 (3.6%)	93,915 (5.1%)	94,512 (5.1%)	
cT4	929 (5.6%)	56,824 (3.1%)	57,753 (3.1%)	
N/R	282 (1.7%)	27,535 (1.5%)	27,817 (1.5%)	
**Nodal staging a diagnosis**				
cN0	12,739 (77.3%)	1,497,912 (81.2%)	1,510,651 (81.2%)	<0.01
cN1+	3,344 (20.3%)	310,954 (16.9%)	314,298 (16.9%)	
N/R	399 (2.4%)	35,913 (1.9%)	36,312 (1.9%)	
**Metastatic stage at diagnosis**				
cM0	16,134 (97.9%)	1,812,741 (98.3%)	1,828,875 (98.3%)	
cM1	12 (0.1%)	1,001 (0.1%)	1,013 (0.1%)	<0.01
N/R	336 (2.0%)	31,037 (1.6%)	31,373 (1.6%)	
**Average tumor size** (IQR) in mm	20.0 (0.0 - 989.0)	16.0 (0.0 - 989.0)	16.0 (0.0 – 989.0)	<0.01
**Average time to treatment** (IQR) in months	22.0 (0.0 – 1111.0)	28.0 (0.0 – 3969.0)	28.0 (0.0 – 3969.0)	<0.01
**Chemotherapy**				
Yes	6,442 (39.1%)	769,366 (41.7%)	775,808 (41.7%)	
No	8,189 (49.7%)	908,756 (49.3%)	916,945 (49.3%)	<0.01
N/R	1,851 (11.2%)	166,657 (9.0%)	168,508 (9.0%)	
**Hormonal therapy**				
Yes	10,477 (63.6%)	1,215,708 (65.9%)	1,226,185 (65.9%)	<0.01
No	4,309 (26.1%)	470,690 (25.5%)	474,999 (25.5%)	
N/R	1,696 (10.3%)	158,381 (8.6%)	8160,077 (8.6%)	
**Radiation Therapy**				
Neoadjuvant RT	54 (0.3%)	5,550 (0.3%)	5,604 (0.3%)	
Adjuvant RT	5,671 (34.4%)	1,038,902 (56.3%)	1,044,572 (56.3%)	
No RT	10,162 (61.7%)	740,683 (40.2%)	750,845 (40.2%)	<0.01
Both neo/adjuvant	4 (0.0%)	772 (0.0%)	776 (0.0%)	
N/R	591 (3.6%)	58,873 (3.2%)	59,464 (3.2%)	
**Year of diagnosis**				
2004	424 (2.6%)	47,143 (2.5%)	47,567 (2.5%)	
2005	426 (2.6%)	51,220 (2.8%)	51,646 (2.8%)	
2006	509 (3.1%)	55,473 (3.0%)	55,982 (3.0%)	
2007	627 (3.8%)	67,631 (3.7%)	68,258 (3.7%)	0.16
2008	935 (5.7%)	104,756 (5.7%)	105,691 (5.7%)	
2009	1,040 (6.3%)	121,772 (6.6%)	122,812 (6.6%)	
2010	1,251 (7.6%)	129,358 (7.0%)	130,609 (7.0%)	
2011	1,222 (7.4%)	137,622 (7.5%)	138,844 (7.5%)	
2012	1,292 (7.8%)	143,376 (7.8%)	144,668 (7.8%)	

2013	1,341 (8.1%)	151,903 (8.2%)	153,244 (8.2%)	
2014	1,395 (8.5%)	157,973 (8.6%)	159,368 (8.6%)	
2015	1,518 (9.2%)	164,281 (8.9%)	165,799 (8.9%)	
2016	1,514 (9.2%)	168,614 (9.1%)	170,128 (9.1%)	
2017	1,543 (9.4%)	173,852 (9.4%)	175,395 (9.4%)	
2018	1,445 (8.8%)	169,805 (9.2%)	171,250 (9.2%)	

IQR: interquartile range; N/R: not reported; ER: estrogen receptor; PR: progesterone receptor; TNBC: Triple negative breast cancer; HER2: human epidermal growth factor receptor 2; cT1: clinical staging T1; cN0: clinical nodal stage 0; cM0: no distant metastasis via clinical assessment; cM1: distant metastasis is present via clinical assessment; RT: radiation therapy.

Supplementary Table S2. Demographic and Clinical Characteristics of IV population by Gender

Characteristics	Male (n=1680)	Female (n=119,217)	Overall (N=120,897)	P-value
**Age at diagnosis** Median (IQR), years	65.0 (23.0 - 90.0)	62.0 (18.0 - 90.0)	62.0 (18.0 - 90.0)	<0.01
**Race**				
White	1,296 (77.1%)	92,664 (77.7%)	93,960 (77.7%)	
Black	311 (18.5%)	20,427 (17.2%)	20,738 (17.2%)	
Asian	21 (1.3%)	2,591 (2.2%)	2,612 (2.2%)	0.04
Other	32 (1.9%)	2,432 (2.0%)	2,464 (2.0%)	
N/R	20 (1.2%)	1,103 (0.9%)	1,123 (0.9%)	
**Insurance status**				
Uninsured	89 (5.3%)	6,176 (5.2%)	6,265 (5.2%)	
Private	532 (31.7%)	46,165 (38.7%)	46,697 (38.6%)	
Governmental	1,015 (60.4%)	63,921 (53.6%)	64,936 (53.7%)	<0.01
N/R	44 (2.6%)	2,955 (2.5%)	2,999 (2.5%)	
**ER Status**				
Negative	212 (12.6%)	27,501 (23.1%)	27,713 (22.9%)	<0.01
Positive	1,343 (80.0%)	80,750 (67.7%)	82,093 (67.9%)	
N/R	125 (7.4%)	10,966 (9.2%)	11,091 (9.2%)	
**PR status**				
Negative	398 (23.7%)	42,666 (35.8%)	43,064 (35.6%)	
Positive	1,135 (67.6%)	64,115 (53.8%)	65,250 (54.0%)	<0.01
N/R	147 (8.7%)	12,436 (10.4%)	12,583 (10.4%)	
**HER2 status**				
Negative	868 (51.7%)	56,380 (47.3%)	57,248 (47.4%)	<0.01
Positive	229 (13.6%)	18,810 (15.8%)	19,039 (15.7%)	
N/R	583 (34.7%)	44.027 (36.9%)	44,610 (36.9%)	

**Subtype**				
TNBC	89 (5.3%)	10,702 (9.0%)	10,791 (9.0%)	
HER2+	229 (13.6%)	18,810 (15.7%)	19,039 (15.7%)	<0.01
ER/PR+ and HER2-	772 (46.0%)	45,317 (38.1%)	46,089 (38.1%)	
N/R	590 (35.1%)	44,388 (37.2%)	44,978 (37.2%)	
**Grade**				
Well differentiated	60 (3.5%)	6,937 (5.7%)	6,997 (5.7%)	
Moderately differentiated	566 (34.0%)	36,258 (30.7%)	36,824 (30.7%)	<0.01
Poorly differentiated	593 (35.2%)	42,376 (35.8%)	42,969 (35.8%)	
Undifferentiated	13 (0.7%)	656 (0.6%)	669 (0.6%)	
N/R	448 (26.6%)	32,194 (27.2%)	32,627 (27.2%)	
**Tumor stage at diagnosis**				
cT1	179 (10.7%)	13,672 (11.5%)	13,851 (11.5%)	
cT2	535 (31.9%)	29,164 (24.5%)	29,699 (24.5%)	<0.01
cT3	133 (7.9%)	15,114 (12.6%)	15,247 (12.6%)	
cT4	488 (29.0%)	35,733 (30.0%)	36,221 (30.0%)	
N/R	345 (20.5%)	25,534 (21.4%)	25,879 (21.4%)	
**Nodal staging at diagnosis**				
cN0	433 (25.7%)	26,906 (22.6%)	27,339 (22.6%)	<0.01
cN1+	947 (56.4%)	69,805 (58.5%)	70,752 (58.5%)	
N/R	300 (17.9%)	22,506 (18.9%)	22,806 (18.9%)	
**Metastatic stage at diagnosis**<br>cM0<br>cM1<br>N/R	22 (1.3%)<br>1,644 (97.9%)<br>14 (0.8%)	2,101 (1.8%)<br>116,085 (97.3%)<br>1,031 (0.9%)	2,123 (1.8%)<br>117,729 (97.3%)<br>1,045 (0.9%)	0.36
**Average tumor size**<br>(IQR) in mm	35.0 (0.0 - 960.0)	39.0 (0.0 - 989.0)	39.0 (0.0 – 989.0)	<0.01
**Average time to treatment**<br>(IQR) in months	17.0 (0.0 – 931.0)	19.0 (0.0 – 3699.0)	19.0 (0.0 – 3699.0)	<0.01
**Chemotherapy**				
Yes	749 (44.6%)	64,469 (54.1%)	65,218 (54.0%)	<0.01
No	721 (42.9%)	40,767 (34.2%)	41,488 (34.3%)	
N/R	210 (12.5%)	13,981 (11.7%)	14,191 (11.7%)	
**Hormonal therapy**<br>Yes<br>No<br>N/R	985 (58.6%)<br>594 (35.4%)<br>101 (6.0%)	60,238 (50.5%)<br>50,899 (42.7%)<br>8,080 (6.8%)	51,493 (42.6%)<br>61,223 (50.6%)<br>8,181 (6.8%)	<0.01
**Radiation Therapy**				
Neoadjuvant RT	18 (1.1%)	1,653 (1.4%)	1,671 (1.4%)	<0.01
Adjuvant RT	255 (15.1%)	13,308 (11.2%)	13,563 (11.2%)	
No RT	1,327 (79.0%)	98,489 (82.6%)	99,816 (82.6%)	
Both Neo/adjuvant RT	6 (0.4%)	271 (0.2%)	277 (0.2%)	
N/R	74 (4.4%)	5,496 (4.6%)	5,570 (4.6%)	
**Year of diagnosis**				

2004	41 (2.4%)	4,560 (3.8%)	4,601 (3.8%)	0.03
2005	48 (2.9%)	4,690 (3.9%)	4,738 (3.9%)	
2006	80 (4.8%)	4,875 (4.1%)	4,955 (4.1%)	
2007	70 (4.2%)	5,447 (4.6%)	5,517 (4.6%)	
2008	89 (5.3%)	6,757 (5.7%)	6,846 (5.7%)	
2009	113 (6.7%)	7,389 (6.2%)	7,502 (6.2%)	
2010	113 (6.7%)	7,779 (6.5%)	7,883 (6.5%)	
2011	110 (6.5%)	8,349 (7.0%)	8,459 (7.0%)	
2012	114 (6.8%)	8,679 (7.3%)	8,793 (7.3%)	
2013	139 (8.3%)	9,168 (7.7%)	9,307 (7.7%)	
2014	132 (7.9%)	9,744 (8.2%)	9,876 (8.2%)	
2015	155 (9.2%)	9,796 (8.2%)	9,951 (8.2%)	
2016	144 (8.6%)	10,400 (8.7%)	10,544 (8.7%)	
2017	157 (9.3%)	10,815 (9.1%)	10,972 (9.1%)	
2018	175 (10.4%)	10,778 (9.0%)	10,953 (9.0%)	

IQR: interquartile range; N/R: not reported; ER: estrogen receptor; PR: progesterone receptor; TNBC: Triple negative breast cancer; HER2: human epidermal growth factor receptor 2; cT1: clinical staging T1; cN0: clinical nodal stage 0; cM0: no distant metastasis via clinical assessment; cM1: distant metastasis is present via clinical assessment; RT: radiation therapy.

Supplemental Table S3. Survival analysis in stage I-III subgroup and by tumor subtypes

Cohort	Gender	Survival rate		*P*-value	Univariate[a]	Multivariate[b]	*P*-value
		**5 yr. (95%CI)**	**10 yr. (95% CI)**		**HR# (95%CI)**	**HR# (95%CI)**	
Stage I-III	Female	0.86 (0.86, 0.86)	0.72 (0.72, 0.73)	<0.0001	ref	ref	<0.0001
	Male	0.77 (0.76, 0.77)	0.57 (0.56, 0.58)		1.76 (1.71-1.81)	2.91 (2.90-2.92)	
TNBC	Female	0.77 (0.77, 0.78)	0.67 (0.66, 0.67)	0.005	ref	ref	0.09
	Male	0.71 (0.66, 0.75)	0.67 (0.62, 0.72)		1.28 (1.08-1.52)	1.01 (0.99-1.03)	
HER2+ BC	Female	0.88 (0.88, 0.88)	0.76 (0.76, 0.76)	<0.001	ref	ref	<0.0001
	Male	0.77 (0.75, 0.80)	0.60 (0.55, 0.65)		1.95 (1.76-2.17)	1.70 (1.68-1.73)	
HR+/HER2-BC	Female	0.88 (0.88, 0.88)	0.74 (0.73, 0.74)	<0.001	ref	ref	<0.0001
	Male	0.78 (0.77, 0.79)	0.58 (0.56, 0.60)		1.95 (1.87-2.03)	0.84 (0.83-0.84)	

Note: TNBC; Triple negative breast cancer, HR; hormone receptor, CI; confidence interval, HR#; Hazards ratio, [a] univariate logistic regression, [b] propensity weighted cox regression model.

Supplemental Table S4. Survival analysis in stage IV subgroup and by tumor subtypes

Cohort	Gender	Survival rate		*P*-value	Univariate[a]	Multivariate[b]	*P*-value
		**5 yr. (95%CI)**	**10 yr. (95% CI)**		**HR# (95%CI)**	**HR# (95%CI)**	
Stage IV	Female	0.28 (0.27, 0.28)	0.13 (0.13, 0.13)	<0.001	ref	ref	<0.0001
	Male	0.23 (0.21, 0.25)	0.08 (0.06, 0.11)		1.13 (1.07-1.19)	1.15 (1.14-1.16)	
TNBC	Female	0.13 (0.12, 0.14)	0.09 (0.08, 0.09)	0.001	ref	ref	<0.0001

	Male	0 (0.02, 0.14)	0 (0.02, 0.14)		1.70 (1.36-2.13)	1.91(1.85-1.98)	
HER2+ BC	Female	0.41 (0.40, 0.41)	0.25 (0.24, 0.26)	0.001	ref	ref	<0.0001
	Male	0.30 (0.75, 0.80)	0.10 (0.01, 0.29)		1.31(1.12-1.55)	1.28 (1.25-1.32)	
HR+/HER2-BC	Female	0.32 (0.32, 0.33)	0.14 (0.13, 0.14)	0.02	ref	ref	<0.0001
	Male	0.27 (0.23, 0.30)	0.14 (0.10, 0.18)		1.11 (1.02-1.22)	1.10 (1.08-1.12)	

Note: TNBC; Triple negative breast cancer, HR; hormone receptor, CI; confidence interval, HR[#]; Hazards ratio, [a] univariate logistic regression, [b] propensity weighted cox regression model.
